# Prevalence and patterns of over-the-counter analgesic use among health sciences students in Aden, Yemen: A cross-sectional study

**DOI:** 10.1016/j.dialog.2026.100361

**Published:** 2026-10-06

**Authors:** Saba A. Mohammed, Samira A. Mahmood, Fekry A. Abdulwali, Afnan T. Mohidden, Muneer M. Hasan, Mugahed F. Mohammed, Muaadh A. Mohammed, Abdulsalam Alhalmi, Khaled S. Ali, Adel A.M. Saeed

**Affiliations:** a Department of Pharmacology, Faculty of Pharmacy, University of Aden, Aden, Yemen; b Department of Pharmaceutics, Faculty of Pharmacy, University of Aden, Aden, Yemen; c Department of Pharmaceutical Chemistry, Faculty of Pharmacy, University of Aden, Aden, Yemen; d Department of Chemistry, Faculty of Science, University of Aden, Aden, Yemen

**Keywords:** Analgesics, Self-medication, Prevalence, Students, Yemen

## Abstract

**Objective:**

The unsupervised use of over-the-counter analgesics constitutes a significant public health concern, particularly in environments with limited regulatory oversight. This study assessed the prevalence, usage patterns, and unadjusted associations of over-the-counter analgesic use among health sciences students in Aden, Yemen.

**Methods:**

A cross-sectional study was conducted between December 2024 and January 2025, enrolling 422 randomly selected students from medicine, pharmacy, dentistry, medical laboratory sciences, and nursing. Data were collected via a structured, pilot-tested, self-administered questionnaire. Descriptive statistics, chi-square, and Fisher's exact tests were applied; all inferential analyses were unadjusted.

**Results:**

The six-month prevalence of over-the-counter analgesic use was 88.9%. Medical Laboratory Sciences students demonstrated the highest observed prevalence (100.0%), whereas nursing students exhibited the lowest (71.4%). Headache was the most frequently cited reason (48.8%). Paracetamol was the most utilised analgesic (74.7%), and community pharmacies served as the primary source (70.9%). Self-reported gastric upset (42%) and vomiting (17%) were common. Fisher's exact test revealed no significant association between faculty and use (*p* = 0.179), whereas sex was significantly associated with reasons for use (*p* = 0.004).

**Conclusions:**

Over-the-counter analgesic use was highly prevalent and predominantly driven by health-related complaints, though study-related motivations were reported. Targeted educational interventions and research investigating local dispensing practices are recommended.

## Highlights

• High prevalence of unsupervised analgesic use among health sciences students.
• Headache emerged as the primary self-reported indication for analgesic use.
• Paracetamol and community pharmacies dominated usage and sourcing patterns.
• Non-medical, study-related motivations were identified among a subset of users.
• Associations relating to faculty and sex remained strictly unadjusted.

## Graphical abstract

Schematic summary of a cross-sectional study of 422 health sciences students in Aden, Yemen (December 2024 to January 2025). Six-month prevalence of over-the-counter analgesic use was 88.9% (375/422). Headache was the leading reported reason (48.8%), non-medical study-related reasons accounted for 6.4%, paracetamol alone was the most common product (74.7%), and pharmacies were the primary source (70.9%); codeine-containing combinations were reported by 33.3% of users. Gastric upset was the most frequent self-reported symptom following use (42%, among 375 users). Associations are unadjusted and symptoms were not clinically verified. The schematic illustration was created with the assistance of a generative AI/design tool and verified by the authors.

**Figure:**
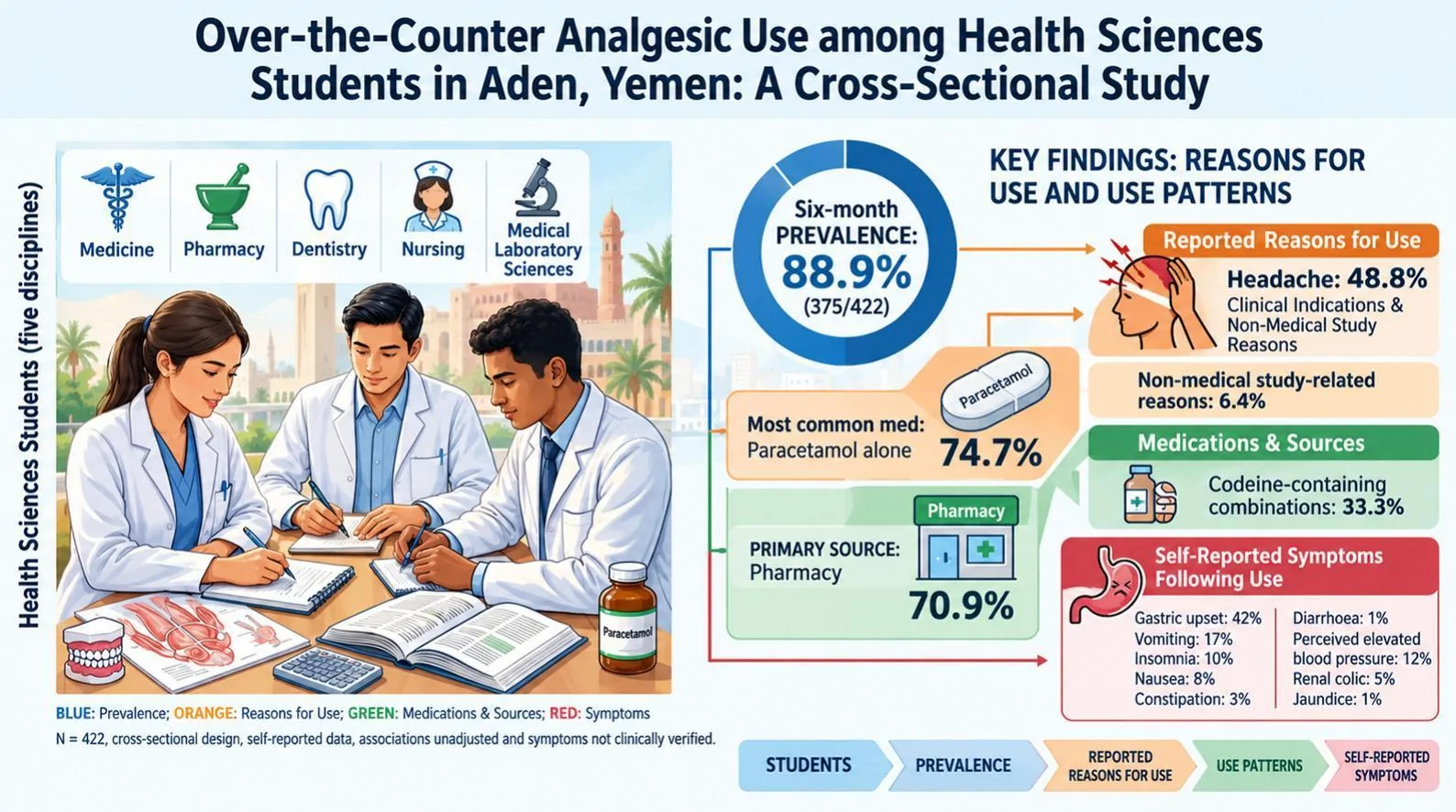

## 1 Introduction

Over-the-counter (OTC) analgesics are medications commonly utilised to manage pain, fever, and mild inflammatory conditions without the requirement of a physician's prescription. Prominent examples include paracetamol, ibuprofen, aspirin, and various other non-steroidal anti-inflammatory drugs (NSAIDs) [1]. Owing to their accessibility, affordability, and convenience, these medications see widespread use across diverse populations, particularly among young adults and university students. Although generally considered safe when used appropriately, inappropriate practices—including incorrect dosing, prolonged consumption, medication sharing, and the utilisation of leftover household medicines—can increase the risk of adverse outcomes and delay necessary medical consultations [2].

The unsupervised use of OTC analgesics represents a critical global public health issue, disproportionately affecting developing and low-income nations where regulatory control over medication dispensing may be constrained [3]. Self-medication behaviours are driven by a complex interplay of contextual factors, such as perceived illness severity, prior experiences with the medication, time limitations, financial constraints, and structural barriers to healthcare access. Within the broader spectrum of OTC medications, analgesics consistently rank as one of the most frequently consumed categories, a trend heavily documented among university student populations [4], [5]. Recent systematic reviews and global epidemiological data confirm a persistently high prevalence of analgesic self-medication among university students and young adults across multiple regions, underscoring the ongoing public health relevance of this practice [6], [7].

Despite common perceptions of their benign nature, the inappropriate use of analgesics can precipitate clinically significant consequences. These include medication errors, adverse drug interactions, gastrointestinal complications, hepatotoxicity, nephrotoxicity, and the delayed diagnosis of severe underlying conditions [8], [9]. Furthermore, the proliferation of easily accessible, yet often unverified, medication-related information via the internet and mass media may actively contribute to the normalisation of self-medication behaviours among highly educated demographic groups [10], [11].

Within university environments, unique stressors such as academic demands, prolonged study hours, and a driven desire to maintain peak concentration or academic performance have been cited as non-medical motivations for analgesic consumption [12]. Such behaviours raise substantive concerns regarding irrational pharmacological practices and the normalisation of unsupervised medication use among students [13]. This concern is perhaps most acute among students enrolled in health sciences disciplines. While it is generally assumed that these students possess superior pharmacological knowledge, their heightened confidence in self-diagnosis, coupled with easier access to medical resources, may paradoxically increase their susceptibility to inappropriate self-medication [14].

In resource-limited healthcare ecosystems, self-medication practices are frequently compounded by systemic challenges, including restricted access to clinical services, widespread financial barriers, severe shortages of healthcare personnel, and fragile regulatory oversight mechanisms [15]. These systemic vulnerabilities have been worsened by prolonged sociopolitical disruptions, potentially driving a heavier reliance on self-care and non-prescription pharmacology. Extant evidence indicates that paracetamol remains a heavily utilised analgesic in these settings, and inappropriate medication habits among young adults represent an emerging public health priority [16].

Although a robust body of literature examines self-medication practices among university students globally, localised evidence remains scarce in certain socio-geographical contexts. Investigating OTC analgesic use among health sciences students provides a critical lens for understanding medication-use behaviours in environments defined by both healthcare system fragility and limited regulatory enforcement. To the best of our knowledge, granular data concerning the unsupervised use of OTC analgesics among health sciences students in Aden, Yemen, remains largely undocumented.

Consequently, this study aimed to quantify the prevalence and map the patterns of OTC analgesic use among health sciences students, identify the most commonly utilised pharmaceutical agents, and examine unadjusted associations between specific sociodemographic characteristics and analgesic consumption. Ultimately, these findings are intended to promote rational analgesic use, inform targeted educational interventions, and advocate for safer medication practices among the next generation of healthcare professionals.

## 2 Methods

### 2.1 Study design and population

An institution-based, descriptive cross-sectional study was executed among undergraduate students enrolled in medical and health-related specialties at the University of Aden. Data collection occurred between December 2024 and January 2025.

### 2.2 Inclusion and exclusion criteria

Eligible participants comprised undergraduate students actively registered in the faculties of medicine, pharmacy, dentistry, medical laboratory sciences, or nursing during the study period. Students who were physically present during the data collection sessions and provided informed consent were included. Those who declined participation, were absent, or submitted incomplete questionnaires were strictly excluded from the analysis.

### 2.3 Sample size and sampling technique

The required sample size was computed using the single population proportion formula: n = Z^2^p(1-p)/d^2^ where ‘n’ represents the necessary sample size, ‘Z' is the standard normal deviate associated with a 95% confidence level (1.96), ‘p’ is the estimated prevalence, and ‘d’ denotes the accepted margin of error (0.05).

A prevalence rate of 80% (*p* = 0.80) was adopted based on recent regional literature documenting high rates of OTC medication use and self-medication among university cohorts in comparable settings, notably Orayj et al. [18] and Al-Worafi et al. [16]. Using these parameters (Z = 1.96, *p* = 0.80, and d = 0.05), the minimum calculated sample size was 246 students. To guarantee robust statistical representation across all academic faculties and to buffer against potential non-responses or incomplete data, the target sample size was elevated to 422 students.

A proportional sampling strategy was utilised to allocate participant quotas to each specialty based on total faculty registration numbers. Participants were subsequently recruited utilising a simple random sampling technique derived from official university registration registries. Every eligible student was assigned a unique numerical identifier, and selections were made via a lottery-based randomization protocol. This methodology ensured that all eligible students possessed an equal probability of selection, independent of sex or academic year.

A total of 422 selected students were approached during scheduled academic sessions. Participation was entirely voluntary; it was explicitly communicated that opting out would carry no punitive consequences regarding academic assessments or faculty relationships. All 422 approached students agreed to participate, yielding a 100% response rate for the targeted cohort. No selected student declined, and no participant was excluded post-distribution based on eligibility criteria. Consequently, 422 questionnaires were incorporated into the final dataset.

The instrument was strictly anonymous, collecting no names, university identification numbers, or alternative identifying metrics. Participants completed the forms independently, and documents were collected in a controlled manner designed to guarantee absolute response confidentiality.

### 2.4 Instrument and data collection

Data were acquired using a structured, pilot-tested, self-administered questionnaire, adapted from previously published instruments used in prior peer-reviewed studies assessing OTC analgesic practices among health sciences cohorts [4]. Initially drafted in English, the questionnaire was translated into Arabic. To verify linguistic fidelity and conceptual equivalence, the Arabic draft was independently back-translated into English.

Face and content validity were appraised by three independent public health specialists who reviewed the instrument for clarity, comprehensiveness, and cultural resonance. While a formal Content Validity Index was not quantified, all expert recommendations were integrated into the final tool. Reliability and operational feasibility were evaluated via a pilot study of 40 students (who were subsequently excluded from the primary analysis). Because the questionnaire measured heterogeneous behavioural and categorical variables rather than a unified latent construct, internal consistency was not treated as a proxy for unidimensional validity. The pilot phase primarily verified item clarity, completion timing, and logistical feasibility.

Data collection occurred face-to-face within lecture halls, following explicit authorization from course instructors. The study's rationale was briefed prior to distribution, with completion requiring approximately five minutes. To mitigate social desirability bias, the process was entirely anonymized.

The questionnaire comprised three core sections:

1. Sociodemographic parameters: age, sex, marital status, academic faculty, and the presence of underlying chronic conditions.
2. OTC analgesic usage patterns: utilisation over the preceding six months, prescription status, primary rationale for use, specific analgesic types consumed, administration routes, and daily dosages.
3. Practices related to analgesic consumption: usage frequency, post-consumption symptom occurrence, symptom typology, instruction-reading habits, and acquisition sources. Multiple-response formats were utilised where appropriate.

The complete questionnaire is available in Supplementary File 1.

### 2.5 Operational definitions

**1. Over-the-counter analgesic use:** Defined as the self-reported consumption of any analgesic formulation without a formal medical prescription during the six months prior to the survey. Data regarding the acquisition source (e.g., community pharmacy, domestic medicine cabinets, family networks, or supermarkets) were collected to accurately map non-prescription behaviours, independent of the formal regulatory classification of the source.

The investigated analgesic formulations encompassed: Panadol (paracetamol/acetaminophen); Panadol Extra (paracetamol/caffeine); Profenid (ketoprofen); Brufen (ibuprofen); Adoflam (diclofenac potassium); Voltaren (diclofenac sodium); Solpadeine (paracetamol/codeine/caffeine); combinations of diclofenac sodium and Solpadeine; alongside a category for other self-reported agents. Codeine-integrated formulations were explicitly isolated due to their availability in the local market without a prescription, regardless of their statutory regulatory status.

**2. Repeated over-the-counter analgesic use:** Characterised as the consumption of a specific analgesic agent on more than three distinct occasions within the preceding six months.

**3. Non-medical use:** Defined as the utilisation of OTC analgesics for outcomes unrelated to pain or pyrexia management, encompassing study-aid concentration, chemically induced wakefulness, relaxation facilitation, or habitual dependency.

**4. Self-reported symptoms following analgesic use:** The self-reported manifestation of any undesirable physiological symptom subsequent to analgesic consumption. Notably, these variables lacked clinical verification, and pharmacological causality was not formally established.

**5. Gastric upset:** Characterised as self-reported epigastric discomfort, dyspepsia, or general stomach distress, categorically separated from incidents of vomiting, diarrhoea, or constipation.

### 2.6 Data analysis

Data were coded, entered, and processed utilising IBM SPSS Statistics (Version 25). Descriptive statistics synthesized the study variables. Continuous data were expressed as means ± standard deviations, while categorical data were articulated as frequencies and percentages. Associations between categorical variables were interrogated using Pearson's chi-square test. Where fundamental assumptions of the chi-square test were violated (specifically, expected cell frequencies <5), Fisher's exact test was applied. Crucially, all inferential statistical analyses were bivariate and entirely unadjusted; no multivariable modelling was executed. The threshold for statistical significance was established at *p* < 0.05. For multiple-response indices, percentages were computed against the appropriate specific denominator, as defined in the relevant table footnotes.

### 2.7 Ethical considerations

The research adhered strictly to the ethical frameworks delineated in the Declaration of Helsinki. Formal ethical clearance was granted by the Ethics Committee of the Faculty of Medicine and Health Sciences, University of Aden (Approval Code: REC-205-2024). Study objectives were communicated clearly prior to data acquisition, and written informed consent was secured from every participant. The protocol emphasized strict voluntarism, guaranteeing the right to withdraw at any juncture without justification. Importantly, it was made clear that neither participation nor withdrawal would negatively impact the students' academic evaluations, grades, or relationships with university faculty. Data were collected anonymously, handled with absolute confidentiality, and utilised exclusively for academic research.

## 3 Results

### 3.1 Sociodemographic characteristics of participants

The final analysis included 422 students. The majority of participants (70.6%) were aged between 18 and 23 years, yielding a mean age of 22.3 ± 2.23 years. Males represented 60.9% of the sample, whereas females accounted for 39.1%. The vast majority of participants were single (90.1%). Regarding faculty distribution, students from the Faculty of Medicine represented the largest cohort (45.3%), followed by Dentistry (23.9%) and Pharmacy (20.6%) (Table 2).

Among the 54 participants reporting chronic health conditions, asthma and “other chronic conditions” were the most prevalent (35.2% each), followed by hypertension (13.0%), dyslipidemia (9.3%), and diabetes mellitus (7.4%) (Table 2).

### 3.2 Prevalence of over-the-counter analgesic use among participants

The overall prevalence of OTC analgesic use during the preceding six months was 88.9%. Across the five faculties evaluated, students from Medical Laboratory Sciences exhibited the highest observed prevalence (100.0%), followed by Dentistry (91.1%), Pharmacy (90.8%), Medicine (87.4%), and Nursing (71.4%) (Table 1). Due to the constrained sample sizes within the Medical Laboratory Sciences and Nursing cohorts, these faculty-specific metrics should be interpreted with appropriate caution.

**Table 1:** Distribution of the study sample and prevalence of over-the-counter analgesic use by faculty (*N* = 422).

Faculty	Total Registered Students	Sample Size	Yes n (%)	No n (%)	Total n (%)
Pharmacy	669	87	79 (90.8)	8 (9.2)	87 (100.0)
Medicine	1462	191	167 (87.4)	24 (12.6)	191 (100.0)
Dentistry	780	101	92 (91.1)	9 (8.9)	101 (100.0)
Medical Laboratory Sciences	164	22	22 (100.0)	0 (0.0)	22 (100.0)
Nursing	154	21	15 (71.4)	6 (28.6)	21 (100.0)
Total	3229	422	375 (88.9)	47 (11.1)	422 (100.0)

Note. Faculty-specific percentages were calculated within each faculty. Total percentages were based on the overall sample (N = 422).

**Table 2:** Sociodemographic characteristics and chronic disease distribution among participants (*N* = 422).

Characteristic	n	%
Age, years		
18 to 23	298	70.6
24 to 29	120	28.5
30 to 35	4	0.9
Mean ± SD	22.3 ± 2.23	
Sex		
Male	257	60.9
Female	165	39.1
Marital status		
Single	380	90.1
Married	36	8.5
Widowed	1	0.2
Divorced	5	1.2
Faculty		
Pharmacy	87	20.6
Medicine	191	45.3
Dentistry	101	23.9
Medical Laboratory Sciences	22	5.3
Nursing	21	4.9
Chronic disease among 54 participants		
Hypertension	7	13.0
Diabetes mellitus	4	7.4
Asthma	19	35.2
Dyslipidemia	5	9.3
Other chronic diseases	19	35.2

Note. Chronic disease percentages were calculated among the 54 participants who reported at least one chronic condition.

**Table 3:** Reasons for over-the-counter analgesic use among participants (*N* = 375).

Category	Reason	n	%
Health-related reasons (*n* = 351, 93.6%)	Headache	183	48.8
	Arthralgia	40	10.7
	Toothache	25	6.7
	Flu or common cold symptoms	28	7.5
	Dysmenorrhea	35	9.3
	Abdominal pain	19	5.1
	Fever	21	5.6
Non-health-related reasons (*n* = 24, 6.4%)	Improving concentration while studying	12	3.2
	Staying awake during study	8	2.1
	Facilitating relaxation or rest	2	0.5
	Psychological habit or dependence	2	0.5

Note. Participants selected one primary reason for over-the-counter analgesic use. Percentages were calculated among the 375 analgesic users.

**Table 4:** Types and frequency of over-the-counter analgesic use among participants (*N* = 375).

Type of Analgesic	Users n (%)	Once n (%)	Twice n (%)	Three Times n (%)	More Than Three Times n (%)
Paracetamol	280 (74.7)	28 (10.0)	31 (11.1)	46 (16.4)	175 (62.5)
Paracetamol + caffeine	155 (41.3)	10 (6.5)	19 (12.3)	16 (10.3)	110 (71.0)
Paracetamol + codeine + caffeine	125 (33.3)	14 (11.2)	12 (9.6)	17 (13.6)	82 (65.6)
Ketoprofen	65 (17.3)	5 (7.7)	6 (9.2)	8 (12.3)	46 (70.8)
Ibuprofen	34 (9.1)	1 (2.9)	2 (5.9)	9 (26.5)	22 (64.7)
Diclofenac potassium	28 (7.5)	3 (10.7)	3 (10.7)	6 (21.4)	16 (57.1)
Diclofenac sodium	65 (17.3)	3 (4.6)	6 (9.2)	8 (12.3)	48 (73.8)
Paracetamol + diclofenac sodium	89 (23.7)	8 (9.1)	7 (8.0)	11 (12.5)	63 (70.5)
Others	14 (3.7)	3 (21.4)	2 (14.3)	0 (0.0)	9 (64.3)

Note. Participants could report more than one type of analgesic; therefore, user percentages exceed 100%. Frequency percentages were calculated within each analgesic type.

**Table 5:** Bivariate associations between selected characteristics and over-the-counter analgesic use and reasons for use.

Part A. Association between selected characteristics and over-the-counter analgesic use (*N* = 422)				
Characteristic	Yes n (%)	No n (%)	Total n (%)	Statistical Test
Age, years				Chi-square = 3.99, *p* = 0.136
18 to 23	260 (87.2)	38 (12.8)	298 (100.0)	
24 to 29	112 (93.3)	8 (6.7)	120 (100.0)	
30 to 35	3 (75.0)	1 (25.0)	4 (100.0)	
Sex				Chi-square = 1.68, *p* = 0.195
Male	224 (87.2)	33 (12.8)	257 (100.0)	
Female	151 (91.5)	14 (8.5)	165 (100.0)	
Marital status				Chi-square = 0.67, *p* = 0.413
Single	337 (88.7)	43 (11.3)	380 (100.0)	
Ever married	38 (90.5)	4 (9.5)	42 (100.0)	
Faculty				Fisher's exact test, *p* = 0.179
Pharmacy	79 (90.8)	8 (9.2)	87 (100.0)	
Medicine	167 (87.4)	24 (12.6)	191 (100.0)	
Dentistry	92 (91.1)	9 (8.9)	101 (100.0)	
Medical Laboratory Sciences	22 (100.0)	0 (0.0)	22 (100.0)	
Nursing	15 (71.4)	6 (28.6)	21 (100.0)	


Part B. Association between reasons for analgesic use and selected characteristics among analgesic users (*N* = 375)				
Characteristic	Health-Related n (%)	Non-Health-Related n (%)	Total n (%)	Statistical Test
Sex				Chi-square = 8.22, *p* = 0.004
Male	203 (90.6)	21 (9.4)	224 (100.0)	
Female	148 (98.0)	3 (2.0)	151 (100.0)	
Faculty				Fisher's exact test, *p* = 0.179
Pharmacy	67 (95.7)	3 (4.3)	70 (100.0)	
Medicine	159 (90.3)	17 (9.7)	176 (100.0)	
Dentistry	88 (95.7)	4 (4.3)	92 (100.0)	
Medical Laboratory Sciences	22 (100.0)	0 (0.0)	22 (100.0)	
Nursing	15 (100.0)	0 (0.0)	15 (100.0)	

a) Note. Pearson chi-square for faculty was 10.44 (p = 0.034); however, Fisher's exact test was utilised for interpretation because expected cell counts fell below 5 in certain faculty groups.

b) Note. Analyses were restricted to participants who reported over-the-counter analgesic use (N = 375). Zero values indicate an absence of responses rather than missing data.

### 3.3 Reasons for over-the-counter analgesic use among participants

Health-related complaints served as the primary rationale for analgesic use among 351 participants, accounting for 93.6% of the 375 total analgesic users. Headache was overwhelmingly the most frequent indication (48.8%), followed by arthralgia (10.7%), dysmenorrhea (9.3%), flu or common cold symptoms (7.5%), toothache (6.7%), fever (5.6%), and abdominal pain (5.1%) (Table 3).

Conversely, 24 participants (representing 6.4%) cited non-health-related motivations. The most prominent among these were cognitive enhancement (improving concentration while studying) (3.2%) and chemically extending study hours (staying awake) (2.1%). Facilitating relaxation and habitual dependency were each reported by a marginal fraction (0.5%) of the cohort (Table 3).

### 3.4 Types and frequency of over-the-counter analgesics used among participants

Paracetamol was the most universally consumed OTC analgesic (74.7%), trailed by paracetamol combined with caffeine (41.3%) and the paracetamol/codeine/caffeine combination (33.3%). Other prevalent regimens included the concomitant administration of paracetamol and diclofenac sodium (23.7%), ketoprofen (17.3%), and diclofenac sodium alone (17.3%). Ibuprofen (9.1%) and diclofenac potassium (7.5%) were utilised with lower frequency (Table 4).

A notable segment of the population engaged in the repeated administration of OTC analgesics over the six-month window. The highest frequency of heavy utilisation (greater than three distinct uses) was associated with diclofenac sodium (73.8%), followed tightly by paracetamol combined with caffeine (71.0%) and ketoprofen (70.8%). Comparable high-frequency patterns were logged for the paracetamol/diclofenac sodium combination (70.5%), the paracetamol/codeine/caffeine blend (65.6%), ibuprofen (64.7%), and paracetamol monotherapy (62.5%). Diclofenac potassium demonstrated the lowest propensity for repeated high-frequency use (57.1%) (Table 4).

### 3.5 Association between sociodemographic characteristics and over-the-counter analgesic use

When stratified by age, students between 24 and 29 years demonstrated the highest prevalence of usage (93.3%). A slightly higher prevalence was noted among females (91.5%) compared to males (87.2%). Analysing marital status revealed absolute prevalence (100.0%) among widowed and divorced students; however, the negligible sizes of these specific subgroups demand highly cautious interpretation. Usage among single and married cohorts stood at 88.7% and 88.9%, respectively.

Regarding academic discipline, Medical Laboratory Sciences (100.0%) and Dentistry (91.1%) recorded the peak prevalence rates, whereas Nursing reported the lowest (71.4%). Bivariate chi-square analysis failed to identify statistically significant associations between analgesic consumption and age, sex, or marital status. While the Pearson chi-square initially flagged a potential association with faculty type (χ^2^ = 10.44, *p* = 0.034), expected cell counts below 5 in certain groups mandated the application of Fisher's exact test. Consequently, the exact-test interpretation confirmed the absence of a statistically significant association (*p* = 0.179) (Table 5).

### 3.6 Association between reasons prompting analgesic use, sex, and faculty type

The overwhelming majority of the cohort relied on analgesics for health-related matters. When segmented by sex, 98.0% of female students cited health-related reasons, contrasting with 90.6% of their male counterparts. From a faculty perspective, all students in Medical Laboratory Sciences and Nursing (100.0%) utilised analgesics for health-related reasons, with similarly high proportions observed in Pharmacy (95.7%), Dentistry (95.7%), and Medicine (90.3%).

Statistical testing confirmed a significant association between biological sex and the primary reason for analgesic use (χ^2^ = 8.22, *p* = 0.004). Conversely, due to small expected cell counts, Fisher's exact test was deployed for faculty type, yielding no statistically significant association (*p* = 0.179) (Table 5).

### 3.7 Source of over-the-counter analgesics among participants

Community pharmacies functioned as the dominant acquisition vector for OTC analgesics (70.9%), followed by preexisting domestic supplies (27.5%), supermarkets (12.3%), and social networks (friends or family members) (10.4%). As multiple responses were permitted, these percentages were calculated strictly against the baseline of the 375 confirmed analgesic users.

### 3.8 Self-reported symptoms following over-the-counter analgesic use among participants

Gastric upset ranked as the most frequently self-reported symptom subsequent to analgesic ingestion (42%), followed by vomiting (17%), insomnia (10%), nausea (8%), constipation (3%), and diarrhoea (1%) (Fig. 1). A minority of participants reported more severe subjective complaints, including perceived elevated blood pressure (12%), renal colic (5%), and jaundice (1%). Crucially, these self-reported symptoms lacked clinical corroboration and must not be conflated with confirmed, causally linked adverse drug reactions.

**Fig. 1:**
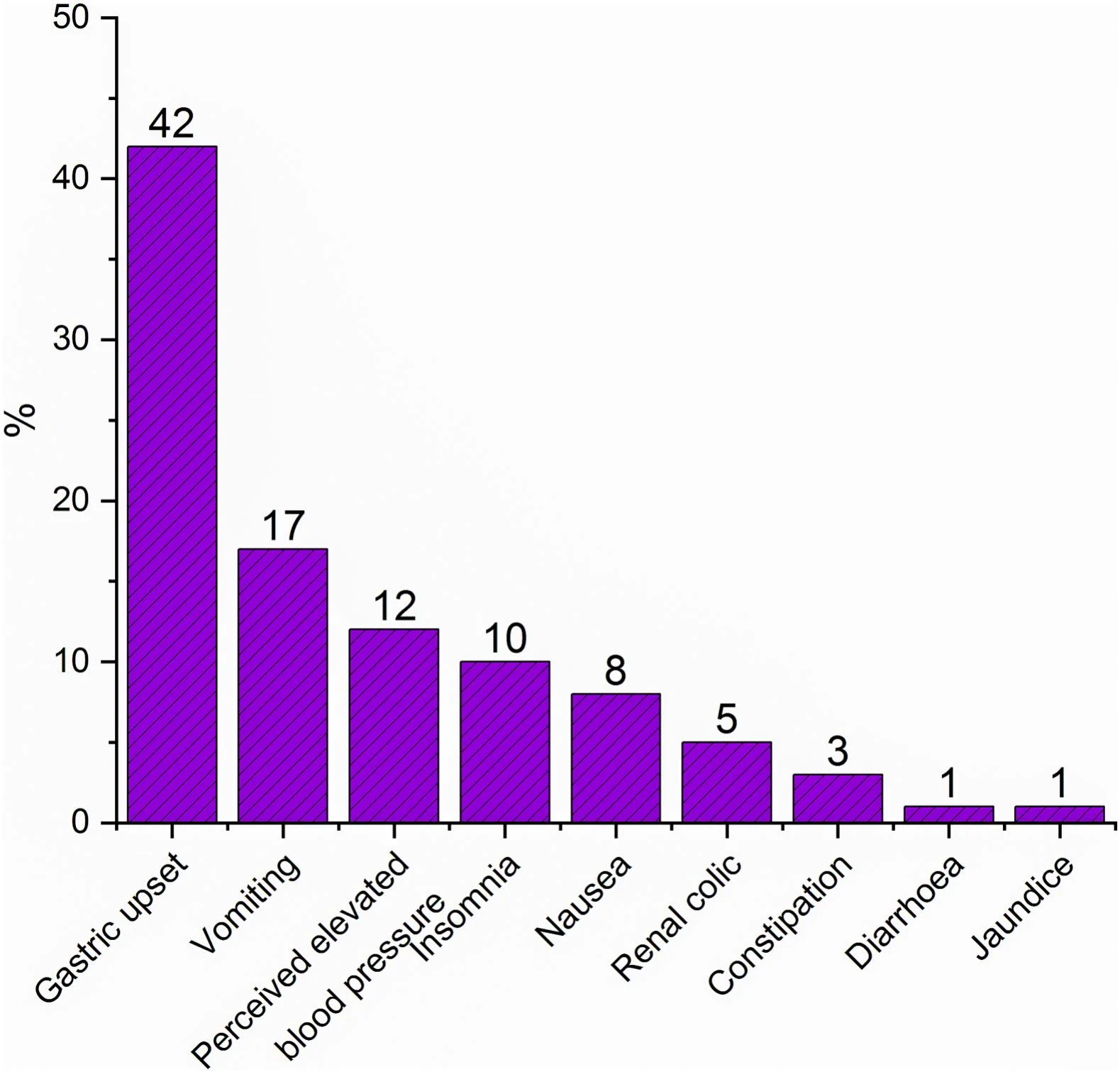
Self-reported symptoms following over-the-counter analgesic use among participants (*N* = 375). Figure note. These symptoms are strictly self-reported and were not clinically verified. They should not be conflated with confirmed adverse drug reactions.

## 4 Discussion

Over-the-counter analgesics remain universally accessible pharmaceutical agents utilised heavily for the autonomous management of pain and minor systemic afflictions [3]. The global increase in their use is particularly pronounced in environments lacking rigorous regulatory frameworks, where unhindered accessibility fosters unsupervised and potentially hazardous consumption patterns [17].

The present study demonstrated a remarkably high prevalence of OTC analgesic usage among health sciences students, with 88.9% reporting consumption within the preceding six months. This finding indicates that analgesic self-medication is highly common in this specific demographic. Concordant prevalence metrics have been documented in parallel settings; Orayj et al. [18] noted an 80% prevalence within Saudi university cohorts, while Patel and Patel [11] identified intense self-medication practices among medical students, specifically pinpointing analgesics as a primary intervention for minor ailments. Discrepancies between specific studies are likely attributable to distinct population demographics, variable healthcare access, divergent cultural paradigms, and nuanced operational definitions of self-medication.

Medication utilisation is influenced by patient experiences, healthcare system factors, and interactions with healthcare providers, which collectively affect medication safety [19]. Recent literature consistently identifies community pharmacists as vital conduits for medication literacy, reinforcing the strategic necessity of pharmacist-led educational and counselling initiatives [20], [21].

The consumption of OTC analgesics by university students is modulated by both somatic and academic factors. Beyond standard physiological complaints (e.g., headache, musculoskeletal discomfort), students sometimes deploy analgesics for non-medical, academically oriented purposes, such as enhancing cognitive focus or chemically forestalling sleep. While the vast majority of our cohort reported health-related motivations, a distinct subset admitted to non-medical usage. It is critical to emphasise that these data represent subjective, self-reported motivations and do not serve as direct, empirical evidence of academic stress, examination anxiety, or chronic sleep deprivation, as these specific psychological constructs were not actively measured. Nonetheless, the presence of non-medical analgesic application highlights an urgent requirement for targeted educational interventions advocating rational pharmacological practices among future healthcare professionals [12], [22], [23].

The interpretation of non-medical motivations, such as ‘staying awake’ or ‘improving concentration,’ requires careful contextualization regarding unmeasured confounders. Specifically, the academic year and prior pharmacological training may introduce a self-censorship bias; senior students with advanced clinical knowledge might underreport non-therapeutic use due to awareness of the hepatotoxic and nephrotoxic risks of unsupervised analgesic consumption. Furthermore, the use of caffeine-containing analgesics may be confounded by dietary caffeine intake and poor sleep hygiene. Consequently, the reported 6.4% prevalence of non-medical use likely represents an absolute minimum, reflecting only those students willing to disclose academically driven coping mechanisms in a supervised classroom environment.

Headache emerged as the dominant indication for analgesic intervention, followed by arthralgia. This aligns seamlessly with established literature positioning headache as the primary driver for analgesic self-medication. For example, Alshahrani et al. [2] found that headache accounted for 50.9% of all analgesic usage among their surveyed student population. Unsurprisingly, paracetamol was the most frequently consumed agent (either isolated or compounded with caffeine or codeine). This finding corroborates global trends identifying paracetamol as the definitive first-line OTC analgesic due to its generally favourable safety profile, profound affordability, and ubiquitous availability [24], [25]. Its proven clinical efficacy against mild-to-moderate pain and pyrexia further cements its status [26].

While variations in analgesic consumption were noted across the different academic disciplines—specifically, peak usage in Medical Laboratory Sciences and minimal usage in Nursing—these trends require cautious interpretation due to restricted subgroup sample sizes. Although these variances might theoretically stem from differing academic burdens or micro-cultural learning environments, these hypotheses were not tested within the current study framework. Therefore, these observations stand strictly as unadjusted associations, rather than confirmed independent variables. The broader literature features similar inconsistencies; certain studies have documented peak prevalence among nursing cohorts, implying that highly localised, contextual variables heavily influence self-medication dynamics [27].

Rigorous statistical modelling (utilising Fisher's exact test to correct for low cell counts) confirmed that faculty type was not significantly associated with analgesic usage, neutralising the initial findings of the Pearson chi-square test. This methodological correction underscores the critical importance of selecting appropriate statistical instruments when evaluating small subgroup clusters. Furthermore, no significant associations linked prevalence to age, sex, or marital status. However, a significant statistical link was identified between biological sex and the underlying motivation for analgesic use, suggesting that gender dynamics may subtly influence the behavioural drivers of self-medication.

A subset of users reported physiological symptoms post-consumption, with gastric upset being the most prevalent complaint. These data must be strictly interpreted as unverified, participant-reported experiences rather than proven adverse drug reactions, as clinical causality was explicitly not investigated. Nevertheless, such complaints remain clinically relevant, because recent cross-sectional studies highlight that young adults frequently underestimate the risk of gastrointestinal and hepatic complications associated with unsupervised NSAID and paracetamol use [9], [28]. The specialised literature emphasizes the pivotal role of pharmacists in identifying pharmaceutical complications and delivering vital counselling [29]. Given the intense prevalence of analgesic utilisation uncovered here, pharmacist-led counselling emerges as a highly logical strategy to foster rational medication usage within university populations [30]. However, because this study did not evaluate localised dispensing practices, regulatory compliance, or the actual frequency of pharmacist interventions, no definitive conclusions can be drawn regarding the current efficacy of regional pharmacy regulations.

### 4.1 Limitations

Several methodological constraints must be considered when interpreting these findings. First, the foundational cross-sectional design inherently precludes the establishment of temporal or causal relationships between sociodemographic traits and analgesic consumption patterns. Second, the reliance on self-reported data over a six-month recall window introduces the risk of recall and reporting biases, specifically regarding usage frequency, motivations, and specific drug choices. Furthermore, because dosages and consumption durations were not clinically verified, true usage intensity may be misclassified or underestimated.

Third, the study was executed exclusively within a single institution, inherently limiting the external validity and generalisability of the findings to different universities or non-health sciences demographics.

Fourth, the methodology of distributing and collecting questionnaires during scheduled, supervised academic sessions introduces the potential for classroom-based response bias. While participation was voluntary, anonymous, and academically unlinked, the physical presence of researchers may have exerted subtle participation pressure or triggered social desirability bias. Consequently, the potential for socially sanitised reporting cannot be entirely dismissed.

Fifth, the constrained sample sizes within certain faculty subgroups (notably Nursing and Medical Laboratory Sciences) eroded the statistical power of those specific subgroup analyses, inflating the uncertainty of those estimates. Additionally, the execution of multiple statistical comparisons elevated the mathematical risk of type I errors (false-positive findings). Because formal adjustments for multiple comparisons were not applied, all statistically significant associations must be viewed as exploratory rather than definitive.

Sixth, the documented post-use symptoms were entirely subjective and unverified. The study lacked the clinical architecture necessary to assess the timing, severity, duration, or causal linkage of these symptoms to specific agents, particularly when participants engaged in polypharmacy or possessed underlying pathologies. Thus, these variables reflect perceived experiences, not confirmed pharmacological reactions.

Several potentially critical confounding variables—such as exact academic year, quantifiable stress indices, and the precise extent of prior pharmacological education—were not measured. Due to the complete absence of multivariable adjustment modelling, the observed bivariate associations may be heavily skewed by unmeasured residual confounding and cannot be classified as independent associations.

Finally, the absence of standardized psychometric instruments to quantify academic burnout, sleep quality, and baseline dietary caffeine intake represents a significant methodological constraint. Without these variables, it is impossible to disentangle whether non-medical analgesic use is an independent coping strategy or merely a surrogate marker for broader lifestyle and sleep-wake cycle disruptions. Future research must incorporate Ecological Momentary Assessment (EMA) to capture real-time motivations and utilize validated scales, such as the Perceived Stress Scale (PSS), to allow for rigorous multivariable adjustment.

## 5 Conclusions and recommendations

This study empirically demonstrates a high prevalence of OTC analgesic usage among health sciences students in Aden, Yemen. Analgesic consumption is overwhelmingly motivated by health-related somatic complaints—predominantly headaches—with paracetamol (both isolated and compounded) functioning as the primary intervention. Following rigorous statistical correction via Fisher's exact test, faculty type was not significantly associated with analgesic usage, whereas biological sex was significantly linked to the underlying motivations for use. No other demographic variables demonstrated significant associations with the prevalence of usage. Community pharmacies served as the primary vector for acquiring these medications.

These findings underscore a pressing demand for targeted educational initiatives designed to promote rational analgesic usage, heighten awareness of potential post-consumption physiological symptoms, and cultivate safe self-medication practices among university cohorts. Future investigative efforts should prioritise the evaluation of local pharmacy dispensing practices, the strictness of regulatory compliance, the frequency of pharmacist counselling, and the measurable impact of educational interventions utilising robust longitudinal or pre-post interventional designs.

## Declaration of generative AI and AI-assisted technologies in the manuscript preparation process

During the preparation of this work the authors used Grammarly and ChatGPT in order to check grammar and improve readability. After using this tool or service, the authors reviewed and edited the content as needed and take full responsibility for the content of the published article.

## CRediT authorship contribution statement

**Saba A. Mohammed:** Writing – original draft, Methodology, Investigation, Conceptualization. **Samira A. Mahmood:** Investigation, Data curation. **Fekry A. Abdulwali:** Investigation, Data curation. **Afnan T. Mohidden:** Investigation, Data curation. **Muneer M. Hasan:** Investigation, Data curation. **Mugahed F. Mohammed:** Investigation, Data curation. **Abdulsalam Alhalmi:** Software, Formal analysis. **Khaled S. Ali:** Validation, Formal analysis. **Adel A.M. Saeed:** Writing – review & editing, Supervision, Conceptualization.

## Funding

This research did not receive any specific grant from funding agencies in the public, commercial, or not-for-profit sectors.

## Declaration of competing interest

The authors declare that they have no known competing financial interests or personal relationships that could have appeared to influence the work reported in this paper.

## Data Availability

Data will be made available on request.
