# Supplementary material for: Prevalence and patterns of over-the-counter analgesic use among health sciences students in Aden, Yemen: A cross-sectional study
Source: Dialogues Health. 2026 Oct 6;9:100361. doi: 10.1016/j.dialog.2026.100361 (PMC13676697; doi:10.1016/j.dialog.2026.100361)
Supplement: Supplementary file 1 [file mmc1.docx]

**SUPPLEMENTARY FILE 1**

**Supplementary File 1: Full Data Collection Questionnaire (verbatim reproduction of the administered instrument)**

**Note to readers and reviewers (editorial note; this note is not part of the administered instrument)**

**Verbatim reproduction.** This supplement reproduces the data collection instrument exactly as administered to participants between December 2024 and January 2025. No item, response option, skip instruction, or response format has been added, removed, reordered, or reworded, and no data were re-analysed or altered in preparing this supplement. Only artefacts introduced during PDF text extraction (for example, broken words within section headings and missing checkbox glyphs) have been normalised or restored to match the administered layout.

**Title wording.** The administered instrument carries the original working title referring to "Medical Students". The sampled frame was nonetheless defined by Section A, item 4, which lists the five participating disciplines, namely pharmacy, medicine, dentistry, medical laboratory sciences, and nursing. The manuscript title and terminology were revised to "health sciences students" during peer review in response to Editor Comment 1, while the instrument itself is preserved unchanged.

**Terminology harmonisation applied in the manuscript only, with no change to the instrument:**

The heading and items labelled **"adverse effects"** in the administered instrument are reported in the revised manuscript as **"self-reported symptoms following analgesic use"**, in line with Editor Comment 8 and Reviewer 1, Major Comment 1.

The response option labelled **"Gastric ulcer"** captured self-reported upper gastrointestinal discomfort; because no clinical or endoscopic verification was performed, it is reported and defined in the manuscript as **"gastric upset"**, meaning self-reported epigastric discomfort, dyspepsia, or general stomach distress, and is not claimed to represent confirmed ulcer disease.

The option **"Increased blood pressure"** is reported as **"perceived elevated blood pressure"** to make explicit that no objective measurement was undertaken.

Spelling variants present in the administered instrument, such as "Diarrhea", are retained here exactly as administered.

**Response formats.** Section B, item 3 required selection of a single primary reason, either health-related or non-health-related, as printed on the instrument. Section B, item 4, Section C, item 3, and the source item permitted multiple responses. These formats are analysed and footnoted accordingly in Tables 3 to 5 and Figure 1 of the revised manuscript.

**Cross-references.** See Methods sections 2.4 (instrument and data collection) and 2.5 (operational definitions), and the point-by-point response to Editor Comment 5.

**Data Collection Questionnaire**

**Prevalence and Practices of Self-Medication with Analgesics among Medical Students in Aden, Yemen – 2024**

**Introduction**

This questionnaire is designed to assess the prevalence and patterns of analgesic use among health sciences students in Aden, Yemen. It also aims to identify the reasons for analgesic use and to assess common practices associated with the use of these medications.

All information collected will be treated with strict confidentiality and used solely for research purposes. The findings will be reported in aggregate form and will not be presented as individual results.

**Section A: General Characteristics of Participants**

Sex ☐ Male ☐ Female

Age (years) __________ years

Marital Status ☐ Single ☐ Married ☐ Widowed ☐ Divorced

College ☐ Pharmacy Medicine ☐ Dentistry ☐ Medical Laboratory Sciences ☐ Nursing

Do you have any chronic medical condition? ☐ Yes ☐ No

If yes, please specify the chronic condition(s): ☐ Hypertension ☐ Diabetes mellitus ☐ Asthma ☐ Dyslipidemia/ high cholesterol ☐ Other: __________________________

**Section B: Pattern of Analgesic Use**

Have you used any analgesic medication during the past six months? ☐ Yes ☐ No

If yes, please answer the following questions.

How did you obtain the analgesic medication? ☐ With a prescription ☐ Without a prescription

What were the reasons for using analgesic medications? (select one)

A. Health-related reasons ☐ Headache Joint pain ☐ Toothache Common cold ☐ Fever ☐ Menstrual pain ☐ Abdominal pain ☐ Other: __________________________

B. Non-health related reasons ☐ To help me concentrate while studying ☐ Habit/ I am accustomed to using them ☐ To help me stay awake while studying ☐ To help me relax ☐ Other: __________________________

Which analgesic medications have you used during the past six months? You may select more than one option.

| **Analgesic medication** | **Used** | **Route of administration** | **Number of doses per day** |
| --- | --- | --- | --- |
| Panadol (Paracetamol/Acetaminophen) | ☐ | ☐ Oral ☐ Injection ☐ Topical ☐ Suppository | ______ |
| Panadol Extra (Paracetamol + Caffeine) | ☐ | ☐ Oral ☐ Injection ☐ Topical ☐ Suppository | ______ |
| Profenid (Ketoprofen) | ☐ | ☐ Oral ☐ Injection ☐ Topical ☐ Suppository | ______ |
| Brufen (Ibuprofen) | ☐ | ☐ Oral ☐ Injection ☐ Topical ☐ Suppository | ______ |
| Adoflam (Diclofenac potassium) | ☐ | ☐ Oral ☐ Injection ☐ Topical ☐ Suppository | ______ |
| Voltaren (Diclofenac sodium) | ☐ | ☐ Oral ☐ Injection ☐ Topical ☐ Suppository | ______ |
| Solpadeine (Paracetamol + Codeine + Caffeine) | ☐ | ☐ Oral ☐ Injection ☐ Topical ☐ Suppository | ______ |
| Combination of diclofenac sodium and Solpadeine | ☐ | ☐ Oral ☐ Injection ☐ Topical ☐ Suppository | ______ |
| Other (please specify): __________ | ☐ | __________ | ______ |

**Section C: Practices Related to Analgesic Use**

How many times did you use analgesic medications during the past six months? ☐ Once ☐ Twice ☐ Three times ☐ More than three times

Did you experience any adverse effects after using analgesic medications? ☐ Yes ☐ No

If yes, what adverse effect(s) did you experience? You may select more than one option. ☐ Vomiting ☐ Nausea ☐ Gastric ulcer ☐ Fluid retention ☐ Constipation ☐ Diarrhea ☐ Jaundice ☐ Insomnia ☐ Renal colic/ kidney pain ☐ Increased blood pressure ☐ Other: __________________________

Have you ever used analgesic medications without reading the instructions or recommended dosage? ☐ Yes ☐ No

Where did you obtain the analgesic medication? ☐ From a pharmacist ☐ From medication previously kept at home ☐ From a friend or family member ☐ From a supermarket ☐ Other: __________________________
